# Supplementary material for: Presence of HPV with overexpression of p16INK4a protein and EBV infection in penile cancer—A series of cases from Brazil Amazon
Source: PLoS One. 2020 May 6;15(5):e0232474. doi: 10.1371/journal.pone.0232474 (PMC7202603; doi:10.1371/journal.pone.0232474)
Supplement: S1 Protocol — (DOCX) [file pone.0232474.s001.docx]

**S1 PROTOCOL – HPV detection and genotyping method and EBV detection**

**1.1 HPV Detection -** All samples were submitted to generic HPV PCR using the consensus primers (PGMY09/11) which amplifies a 450 bp DNA fragment within the L1 region of mucosal HPV [[1](#_ENREF_1)]. Amplification was carried out as previously described using 50-100 ng of DNA in 25 µL of reaction mixture and a thermocycling profile of 1 cycle at 5 min at 95 °C, followed by 40 cycles: 1 min at 95 °C, 1 min at 55° C, and 1 min at 72 °C, with a final extension for 10 min at 72 °C. The PCR products (450 bp DNA) were analyzed on 1.5 % agarose gel stained with SYBR™ Safe DNA Gel Stain (Invitrogen Life Technologies, São Paulo, Brazil) for visualization of DNA under UV light and 100 bp DNA ladder was used as molecular weight control pattern. Precautions to avoid contamination were followed. DNA from the HeLa cell line which harbors 10-20 copies of integrated HPV 18 per cell was used as a positive control in all reactions.

| **PCR HPV - PGMY09/11** | | | |
| --- | --- | --- | --- |
| **Components** | **Volume (µl)** | **Final concentration** | |
| H_2_O | 14.5 |  |  |
| PCR buffer 10x (without MgCl₂) | 2.5 | 1 | X |
| MgCl₂ (50 mM) | 0.8 | 1.6 | mM |
| dNTP (10 mM) | 0.5 | 0.2 | mM |
| *Primer* PGMY 09/11 (10 µM) | 0.5 | 0.2 | µM |
| *Primer* PCO4 (10 µM) | 0.5 | 0.2 | µM |
| *Primer* GH20 (10 µM) | 0.5 | 0.2 | µM |
| Taq Platinum 5 U/µl | 0.2 |  |  |
| DNA (sample) | 5 |  |  |
| Total (µl) | 25 |  |  |

| ***Primer*** |  | **Sequence (5’ – 3’)** | | | | | | |
| --- | --- | --- | --- | --- | --- | --- | --- | --- |
| **PGMY 11 - A** | **...................** | **GCA** | **CAG** | **GGA** | **CAT** | **AAC** | **AAT** | **GG** |
| **PGMY 11 – B** | **..................** | **GCG** | **CAG** | **GGC** | **CAC** | **AAT** | **AAT** | **GG** |
| **PGMY 11 – C** | **..................** | **GCA** | **CAG** | **GGA** | **CAT** | **AAT** | **AAT** | **GG** |
| **PGMY 11 – D** | **..................** | **GCC** | **CAG** | **GGC** | **CAC** | **AAC** | **AAT** | **GG** |
| **PGMY 11 - E** | **..................** | **GCT** | **CAG** | **GGT** | **TTA** | **AAC** | **AAT** | **GG** |
| **PGMY09 – F** | **..................** | **CGT** | **CCC** | **AAA** | **GGA** | **AAC** | **TGA** | **TC** |
| **PGMY09 – G** | **..................** | **CGA** | **CCT** | **AAA** | **GGA** | **AAC** | **TGA** | **TC** |
| **PGMY09- H** | **..................** | **CGT** | **CCA** | **AAA** | **GGA** | **AAC** | **TGA** | **TC** |
| **PGMY09 – I** | **..................** | **G** | **CCA** | **AGG** | **GGA** | **AAC** | **TGA** | **TC** |
| **PGMY09 – J** | **..................** | **CGT** | **CCC** | **AAA** | **GGA** | **TAC** | **TGA** | **TC** |
| **PGMY09 – K** | **..................** | **CGT** | **CCA** | **AGG** | **GGA** | **TAC** | **TGA** | **TC** |
| **PGMY09 – L** | **..................** | **CGA** | **CCT** | **AAA** | **GGG** | **AAT** | **TGA** | **TC** |
| **PGMY09 – M** | **..................** | **CGA** | **CCT** | **AGT** | **GGA** | **AAT** | **TGA** | **TC** |
| **PGMY09 - N** | **..................** | **CGA** | **CCA** | **AGG** | **GGA** | **TAT** | **TGA** | **TC** |
| **PGMY09 - P** | **..................** | **G** | **CCC** | **AAC** | **GGA** | **AAC** | **TGA** | **TC** |
| **PGMY09 - Q** | **..................** | **CGA** | **CCC** | **AAG** | **GGA** | **AAC** | **TGG** | **TC** |
| **PGMY09 - R** | **..................** | **CGT** | **CCT** | **AAA** | **GGA** | **AAA** | **TGG** | **TC** |
| **HMB01** | **..................** | **GCG** | **ACC** | **CAA** | **TGC** | **AAA** | **TTG** | **GT** |

**1.2. E7 HPV16/HPV18 type-specific real-time PCR**

All samples were also submitted to two specific TaqMan based real-time qPCR assays targeting either HPV16/HPV18 E7 gene in an ABI 7300 Real-Time PCR System (Applied Biosystems, Foster City, CA). All samples and controls were run in duplicate.

**HPV16-E7** qPCR assay included the following primers: forward (5’GATGAAATAGATGGTCCAGC3’) and reverse (5’GCTTTGTACGCACAACCGAAGC3’) primers, and the probe (5’FAM-CAAGCAGAACCGGACAG-MGB-NFQ) in a final reaction volume of 25 μL [[2](#_ENREF_2)]. Each qPCR reaction contained 1X TaqMan master mix (Applied Biosystems, Foster City, CA), 400 nM each of the forward and reverse primers, 200 nM of fluorogenic TaqMan probe, and 50-100 ng of DNA. The amplification conditions consisted of 50 °C for 2 min and 95°C for 10 min, followed by 40 cycles of 95 °C for 15 sec, 55 °C for 1 min, and 60 °C for 1 minute. DNA from a SiHa cell line which contains 1-2 copies of integrated HPV 16 per cell was used as a positive control in all reactions.

| **qPCR HPV 16** | | | |
| --- | --- | --- | --- |
| **Components** | **Volume (µl)** | **Final concentration** | |
| H_2_O | 5 |  |  |
| TaqMan Master Mix 2x | 12.5 | 1 | X |
| P HPV 16 E7 (10 µM) | 1.0 | 0,4 | µM |
| P HPV 16 E7S (10 µM) | 1.0 | 0,4 | µM |
| Probe HPV 16 (10 µM) | 0.5 | 0,2 | µM |
| DNA (sample) | 5 |  |  |
| Total (µl) | 25 |  |  |

**HPV18-E7** qPCR assay included the following primers: forward (5’AAGAAAACGATGAAATAGATGGA3’) and reverse (5’GGCTTCCACCTTACAACACA3’) primers, and a probe (5’VIC-AATCATCAACATTTACCAGCC-MGBNFQ3’) in a final reaction volume of 25 μL [[2](#_ENREF_2)], each qPCR contained 1X TaqMan master mix (Applied Biosystems, Foster City, CA), 400 nM each of the forward and reverse primers, 400 nM fluorogenic TaqMan probe, and 50-100 ng of DNA. The amplification conditions consisted of 50°C for 2 min and 95°C for 10 min, followed by 40 cycles of 95°C for 15 sec, 50°C for 1 min, and 60°C for 1 min. DNA from the HeLa cell line which harbors 10-20 copies of integrated HPV 18 per cell was used as a positive control in all reactions.

| **qPCR HPV 18** | | | |
| --- | --- | --- | --- |
| **Components** | **Volume (µl)** | **Final concentration** | |
| H_2_O | 4.5 |  |  |
| TaqMan Master Mix 2x | 12.5 | 1 | X |
| P HPV 18 E7 (10 µM) | 1.0 | 0.4 | µM |
| P HPV 18 E7S (10 µM) | 1.0 | 0.4 | µM |
| Probe HPV 18 (10 µM) | 1.0 | 0.4 | µM |
| DNA (sample) | 5 |  |  |
| Total (µl) | 25 |  |  |

**1.3 HPV Genotyping - PapilloCheck® HPV-Screening -** All samples that were positive in generic HPV DNA (PGMY09/11) and negative for 16/18 genotypes were submitted to HPV-Screening Test (Greiner Bio-One GmbH, Frickenhausen, Germany) to identify other genotypes. This is a PCR-based DNA microarray system for detection and identification of 24 HPV genotypes, including 16 high-risk HPV genotypes (HPV 16, 18, 31, 33, 35, 39, 45, 51, 52, 56,58, 59, 68, 70, 73, 82), 2 probable high-risk HPV genotypes (HPV 53,66) and 6 low-risk-HPV genotypes (HPV 6, 11, 40, 42, 43, 44/55) [[3](#_ENREF_3)].

**1.4 EBV detection**

EBV DNA detection was performed as described elsewhere [[4](#_ENREF_4)]. Briefly, a sensitive multiplex PCR which amplifies 182 bp within the Exons 4/5 from the terminal protein RNA of EBV and a fragment of human β-actin 450 bp as internal control. The following pairs of primers for PCR amplification were used: EP5-AACATTGGCAGCAGGTAAGC and EM3 -ACTTACCAAGTGTCCATAGGAGC for EBV and B-ACT F–TCTACAATGAGCTGCGTGTG and B-ACT R -CATCTCTTGCTCGAAGTC for β-actin. PCR was performed as follows: 1 cycle at 5 min at 95°C, followed by 10 cycles of 30 sec at 95°C, 60 sec at 63°C and subsequently by 30 cycles of 30 sec at 95°C, 30 sec at 60°C, and 30 sec at 72°C, with a final extension for 40 sec at 72°C. PCR products were analyzed on 1.8% agarose gel stained with SYBR™ Safe DNA Gel Stain (Invitrogen Life Technologies, São Paulo, Brazil) for visualization of DNA under UV light and 100 bp DNA ladder was used as molecular weight control pattern. An EBV positive known sample from the laboratory was used as control.

| **PCR EBV** | | | |
| --- | --- | --- | --- |
| **Components** | **Volume (µl)** | **Final concentration** | |
| H_2_O | 15.3 |  |  |
| PCR buffer 10x (without MgCl₂) | 2.5 | 1 | X |
| MgCl_2_ (25 mM) | 1.5 | 1.5 | mM |
| dNTP (10 mM) | 1.0 | 0.4 | mM |
| EM3/EP5 (10 µM) | 0.75 | 0.3 | µM |
| Bct S/S (10 µM) | 0,75 | 0,3 | µM |
| Taq Platinum 5 U/µl | 0.2 |  |  |
| DNA (sample) | 3 |  |  |
| Total (µl) | 25 |  |  |

**References**

1. Gravitt P, Peyton C, Alessi T, Wheeler C, Coutlee F, et al. (2000) Improved amplification of genital human papillomaviruses. Journal of clinical microbiology 38: 357-361.

2. Veo CA, Saad SS, Fregnani JHT, Scapulatempo-Neto C, Tsunoda AT, et al. (2015) Clinical characteristics of women diagnosed with carcinoma who tested positive for cervical and anal high-risk human papillomavirus DNA and E6 RNA. Tumor Biology 36: 5399-5405.

3. Heard I, Cuschieri K, Geraets D, Quint W, Arbyn M (2016) Clinical and analytical performance of the PapilloCheck HPV-Screening assay using the VALGENT framework. Journal of Clinical Virology 81: 6-11.

4. Markoulatos P, Georgopoulou A, Siafakas N, Plakokefalos E, Tzanakaki G, et al. (2001) Laboratory diagnosis of common herpesvirus infections of the central nervous system by a multiplex PCR assay. Journal of clinical microbiology 39: 4426-4432.
