# Supplementary material for: Presence of HPV with overexpression of p16INK4a protein and EBV infection in penile cancer—A series of cases from Brazil Amazon
Source: PLoS One. 2020 May 6;15(5):e0232474. doi: 10.1371/journal.pone.0232474 (PMC7202603; doi:10.1371/journal.pone.0232474)
Supplement: S2 Protocol — (DOCX) [file pone.0232474.s002.docx]

**S2 PROTOCOL - Immunohistochemical for p16^INK4a^ protein**

**Tissue microarray (TMA) building**

Representative formalin-fixed paraffin–embedded (FFPE) twenty six tumor samples were adequate for tissue microarray (TMA) construction. After pathologist review to confirm the location of tumor for sampling, TMAs were assembled from triplicate 0.6 mm cores of FFPE primary tumor samples using a Beecher Manual Tissue Arrayer (Beecher Instruments, Sun Prairie, WI, USA).

**Immunohistochemistry for p16^INK4a^**

Twenty six samples from PC patients, spotted in tissue microarray were submitted to the immunohistochemistry (IHC) assay for qualitative detection of the p16^INK4a^ (21 samples were not available due to pre-analytical factors). IHC assay was performed in an automated system using the Ventana® BenchMark Ultra according to the manufacturer’s instructions. The IHC slides were analyzed by a pathologist. Positivity for p16^INK4a^ was defined as unequivocally nuclear and cytoplasmic staining of at least 70% of the tumor cells, S1 Fig [[1](#_ENREF_1),[2](#_ENREF_2)].

**S1 Fig. This is the S1 Fig Title.** Examples of p16^INK4a^ Immunohistochemical expression. This is the S1 Fig legend. Patterns of p16 expression in penile carcinomas. Microarray tissue block immunohistochemistry for p16 (from left to right): a. absence; b. strong and diffuse cytoplasmic staining; c. Moderate and focal cytoplasmic staining; d. weak and focal cytoplasmic staining; e. absence of staining; f. strong and diffuse cytoplasmic staining.

**References**

1. Bezerra SM, Chaux A, Ball MW, Faraj SF, Munari E, et al. (2015) Human papillomavirus infection and immunohistochemical p16INK4a expression as predictors of outcome in penile squamous cell carcinomas. Hum Pathol 46: 532-540.

2. Cubilla AL, Lloveras B, Alejo M, Clavero O, Chaux A, et al. (2011) Value of p16INK4a in the pathology of invasive penile squamous cell carcinomas: a report of 202 cases. Am J Surg Pathol 35: 253-261.
