# Supplementary figures and images for: Presence of HPV with overexpression of p16INK4a protein and EBV infection in penile cancer—A series of cases from Brazil Amazon
Source: PLoS One. 2020 May 6;15(5):e0232474. doi: 10.1371/journal.pone.0232474 (PMC7202603; doi:10.1371/journal.pone.0232474)

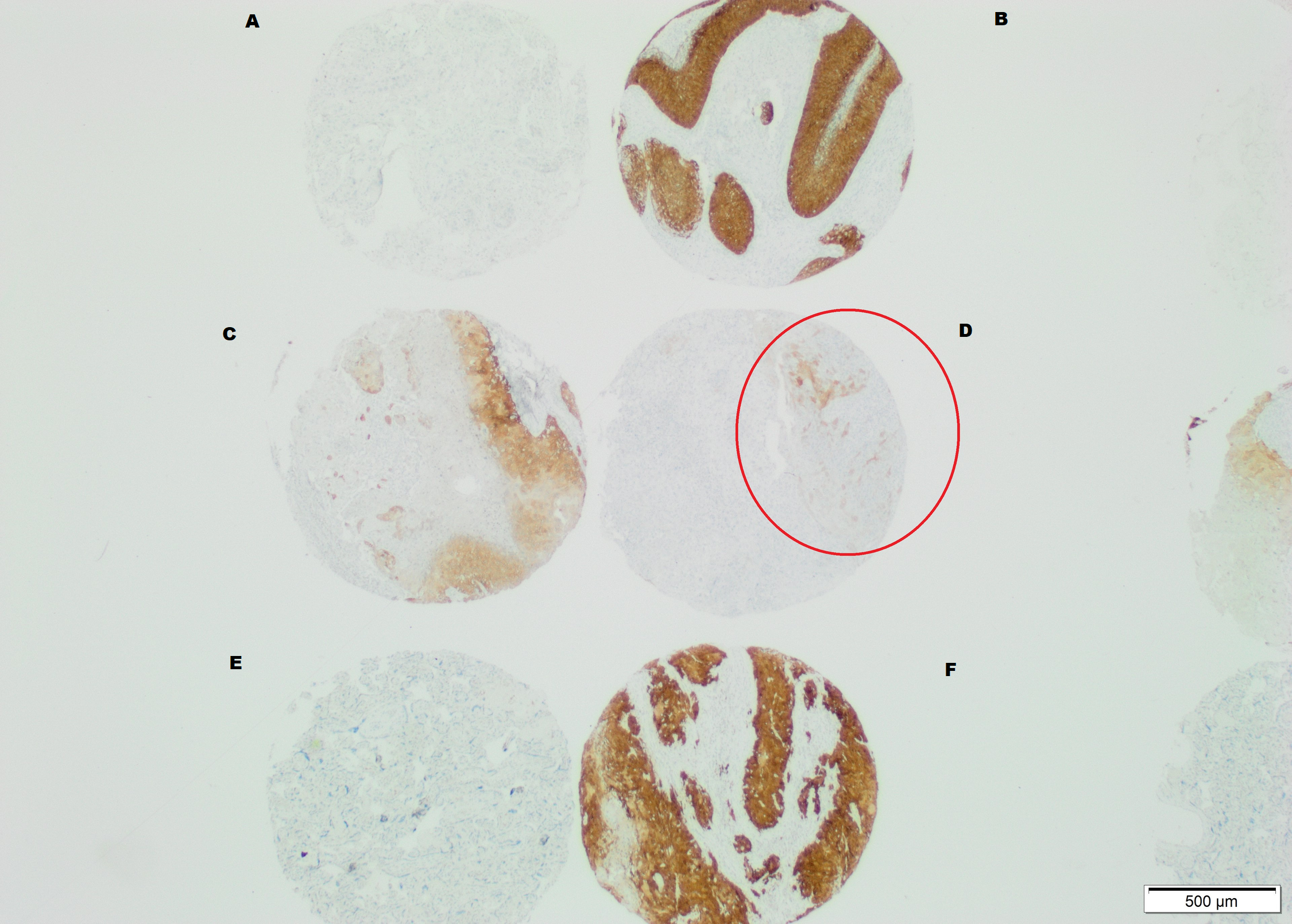

Supplement: S1 Fig — Microarray tissue block immunohistochemistry for p16INK4a (from left to right): a. absence; b. strong and diffuse cytoplasmic staining; c. Moderate and focal cytoplasmic staining; d. weak and focal cytoplasmic staining; e. absence of staining; f. strong and diffuse cytoplasmic staining. (TIF) [file pone.0232474.s003.tif]
