# Supplementary material for: Presence of HPV with overexpression of p16INK4a protein and EBV infection in penile cancer—A series of cases from Brazil Amazon
Source: PLoS One. 2020 May 6;15(5):e0232474. doi: 10.1371/journal.pone.0232474 (PMC7202603; doi:10.1371/journal.pone.0232474)
Supplement: S1 Table — EBV: Epstein-Barr virus; HPV: Human papillomavirus, n: Absolute frequency; +:positive; -:negative. (DOCX) [file pone.0232474.s004.docx]

**S1 TABLE** – Distribution of HPV, EBV status and p16^INK4a^ overexpression among the patients deceased and survived with penile cancer at Amazon - Brazil.

| **Deceased (n=18)** | **Survived (n=29)** |
| --- | --- |
| HPV-/EVB- (n=3) | HPV-/EBV- (8) |
| HPV+/EVB+ (n=1) | - |
| - | HPV-/EBV+ (4) |
| - | HPV+/EBV- (5) |
| p16-/HPV-/EVB- (n=1) | p16-/HPV-/EBV- (3) |
| p16-/HPV-/EVB+ (n=3) | - |
| p16-/HPV+/EVB- (n=3) | p16-/HPV+/EBV- (2) |
| - | p16-/HPV+/EBV+ (2) |
| p16+/HPV-/EVB- (n=1) | p16+/HPV-/EBV- (2) |
| p16+/HPV-/EVB+ (n=1) | - |
| p16+/HPV+/EVB- (n=3) | p16+/HPV+/EBV- (2) |
| p16+/HPV+/EVB+ (n=2) | p16+/HPV+/EBV+ (1) |
