# Supplementary material for: Presence of HPV with overexpression of p16INK4a protein and EBV infection in penile cancer—A series of cases from Brazil Amazon
Source: PLoS One. 2020 May 6;15(5):e0232474. doi: 10.1371/journal.pone.0232474 (PMC7202603; doi:10.1371/journal.pone.0232474)
Supplement: S1 File — (DOCX) [file pone.0232474.s005.docx]

**S1 FILE - DATA COLLECTION INSTRUMENT - PENIS CANCER**

**1. Identification:**

Project patient number: _______________

Date of data collection: __/__/__

Responsible for data collection: ______________________________________________

Medical record number: _______________

Patient Initials: _______________

City where you live: _______________

Address: ___________________________________________________________________________

__________________________________________________________________________________

Address of stay in Manaus (family home that stays, if you are a country patient): ________________

__________________________________________________________________________________

Telephone (always fill in with two contacts): _______________

Date of sample collection (surgical specimen): __/__/__

Laboratory where the biopsy was sent: _______________

**2. Social and Environmental Data:**

2.2 Level of Education

( ) illiterate

( ) Incomplete 1st degree

( ) complete 1st degree

( ) Incomplete high school

( ) complete high school

( ) incomplete higher education

( ) Graduated

2.3 Marital Status

( ) single ( ) married ( ) widowed ( ) divorced ( ) Stable union

2.4 Place of Residence

- Perimeter: ( ) urban ( ) rural

- Number of people residing in the same household: _____________

- Origin (city / state): _______________________________________________

**3. Data Related to the Biology of the Individual:**

3.1. Date of Birth: ____________ / Age at the time of attendance: _______________

3.2 Race: ( ) White ( ) black ( ) brown ( ) indigenous ( ) yellow

3.3. In the family, have there been any other cases of cancer? ( )Yes ( )No

Specify relationship _____________________________________________________

3.4 Previous history of sexually transmitted disease? ( )Yes ( )No

If yes, IST type:

( ) Don't know ( ) Gonorrhea ( ) Syphilis ( ) Urethritis ( ) AIDS ( ) Condyloma acuminata ( ) Others. Please specify: ____________________________.

3.5 Age of first sexual intercourse: _______________________

3.6 Does it have phimosis? ( )Yes ( )No

3.7 Postectomy? ( )Yes ( )No If yes,

3.8 Time of performing the postectomy surgery: ( ) Childhood ( ) Adolescence ( ) Adult life

3.9 Are you currently undergoing treatment for any other type of disease? ( )Yes ( )No

Please specify: ______________________________________________________________________

3.10 Did you have pre-neoplastic diseases? ( )Yes ( )No If yes, which?

( ) Leukoplakias ( ) Balanithixerotic obliterans ( ) Bowen's disease

( ) Bowenoid papulosis ( ) Condyloma acuminata ( ) Queyrat

Other (specify): _____________________________________________

3.11 Age at which the CCEP manifested (years): _________

**4. Lifestyle related data:**

4.1 Do you have or did you have an active sex life? ( )Yes ( )No If yes: When was your last sexual intercourse? _________________________________________________________________

4.2 If you have an active sex life, do you take precautions in relation to the prevention of sexually transmitted diseases? ( )Yes ( )No

4.3 Regarding smoking:

( ) smoker /How long have you smoked? _________ Amount of cigarettes / day? _____________

( ) ex-smoker/How long have you stopped? ________ Amount of cigarettes / day? _____________

( ) non-smoking

4.4 If you are a non-smoker (or ex-smoker), do you share indoor spaces with smokers in your daily life? ( )Yes ( )No

If Yes, Where? ( ) home ( ) work ( ) others. Please specify: ____________________

**5. Sexual History and Zoophilia**

5.1 Number of sexual partners: **(0)** zero **(1)** less than 5 **(2)** 5 a10 **(3)** 10 a15

**(4)** 15 to 20 **(5)** more than 20 **(8)** Not applicable **(9)** Ign ......................................**(____)**

5.2 Sexual relations with prostitutes: **(0)** no **(1)** yes ....................................................................**(____)**

5.3 Sexual relations with animals: **(0)** no **(1)** yes .........................................................................**(____)**

If yes which animal (one or more): **(1)** mare **(2)** mule **(3)** donkey / donkey **(4)** goat **(5)** sheep **(6)** calf **(7)** adult bovine **(8)** buffalo calf **(9)** adult buffalo **(10)** canine **(11)** chicken **(12)** duck **(13)** Others__________________ **(88)** Not applicable **(99)** Ign ..................................................**(____)**

Exposure time: Early age ___________________________

Exposure time: Final age ___________________________

Frequency: **(0)** daily **(1)** alternate days **(2)** 2x week **(3)** 3x week or more **(4)** weekly **(5)** biweekly **(6)** monthly **(7)** bimonthly **(8)** quarterly **(9)** semiannual **(10)** anual **(11)** only once (12) Other ______________ (88) Not applicable (99) Ign ............................................................**(____)**

Practice: **(1)** Individual **(2)** Group **(8)** Not applicable ......................................................................**(____)**

**6. Data Related to Injury Description Clinical data (before surgery):**

6.1 Time elapsed between the appearance of the first signs and symptoms and the demand for health services?

( ) more than six months and less than one year

( ) greater than or equal to one year.

6.2 Date of diagnosis of CCEP: _________________

6.3 Biopsy: ( )yes (has the patient already had a biopsy in the first outpatient consultation?) ( ) No

6.4 Time between the appearance of the lesion and the biopsy (months): ______________________

6.5 Revious treatments before biopsy: ( ) No ( ) Topic ( ) Injectable ( ) Oral

6.6 Biopsy report:

( ) Well-differentiated CEEP ( ) Moderately differentiated CEEP ( ) Undefined CEEP ( ) Carcinoma “in situ” ( ) Verrucous cancer ( ) Sarcoma

6.7 Degree according to biopsy: ( )G I ( )G II ( )G III ( )G IV

6.8 Type of lesion according to the macroscopic classification: ( ) vegetative ( ) ulcerated

6.9 Topographic location of lesions: ( ) glans ( ) foreskin ( ) body ( ) base

( ) glans and foreskin ( ) glans, foreskin and stem ( ) the entire organ

**7. Data based on the analysis of the surgical specimen:**

7.1 Did you perform surgical treatment? ( )Yes ( )No

7.2 If you have undergone surgical treatment, what type of treatment is used?

( ) Partial penectomy ( ) Radical penectomy

7.3 Did you perform inguinal lymphadenectomy? ( ) not ( )early ( )late ( )modified

7.4 Did you perform an iliac lymphadenectomy? ( ) not ( )early ( )late ( )modified

7.5 Result of the biopsy referring to the surgical specimen (whether partial or total penectomy):

( ) Well-differentiated CEEP ( ) Moderately differentiated CEEP ( ) Undefined CEEP ( ) Carcinoma “in situ” ( ) Verrucous cancer ( ) Sarcoma

7.6 TNM staging based on the evaluation of the surgical specimen:

a) PT Staging: ( ) TIS ( )T1 ( )T2 ( )T3 ( )T4

b) PN staging: ( ) N0 ( )N1 ( )N2 ( )N3 ( )NX

c) PM Staging: ( )M0 ( )M1

7.7 Degree after analysis of the surgical specimen: ( )G I ( )G II ( )G III ( )G IV

7.8 Did you perform palliative treatment?

( )Systemic chemotherapy ( )Topical chemotherapy ( ) Radiotherapy

7.9 Postoperative chemotherapy? ( )Yes ( )No

**8. Molecular Diagnostics:**

8.1 HPV ( ) Negative ( ) PGMY ( ) qPCR ( )Paillocheck


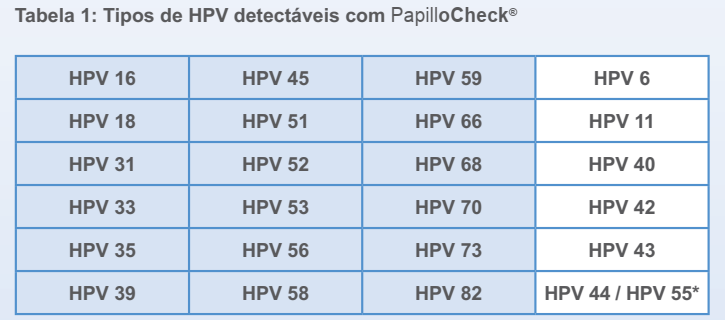


8.2 EBV ( ) Negative ( ) Positive

8.3 p16

( ) Negative ( ) strong positive ( )Moderate Positive ( ) Weak Positive
