# Supplementary material for: Presence of HPV with overexpression of p16INK4a protein and EBV infection in penile cancer—A series of cases from Brazil Amazon
Source: PLoS One. 2020 May 6;15(5):e0232474. doi: 10.1371/journal.pone.0232474 (PMC7202603; doi:10.1371/journal.pone.0232474)
Supplement: Attachment — Submitted filename: response_to_reviewers_AlvesVCR.docx [file pone.0232474.s006.docx]

**Reply to reviewers**

Reviewer #1:

The authors present tumor tissue analyzes from 47 patients with penile cancer. Samples were analyzed for the presence of HPV and EBV DNA by PCR. In addition, p16 INK4a expression was evaluated by immunohistochemistry. The results roughly agree with those in the world literature. In the second sentence of the introduction the authors state: In Brazil, PC accounts for approximately 2.1% of all tumors in men being the highest incidence reported in the world (2.9-6.8 cases per 100,000 men-years). In the cited article, Brazil appears in the first place in the ranking of Latin America being surpassed in the world by Romania and Uganda.

*We agree and have corrected in the texts. (Line 57-58)*

The authors presented a survival curve showing a better prognosis for Ebv- patients when compared to Ebv +. I would like they comment on why this result.

*We agree with the reviewer that there is a trend due to the sudden drop of the survival curve. However, the comparison showed no difference (P_log rank_ = 0.106).*

**Reply to reviewers**

Reviewer #2:

The present study is about penile cancer and oncogenic viruses HPV and EBV and their relationship with clinical and pathological characteristics. The work is important because penile carcinoma is an uncommon neoplasm, although in some regions of the world, such as Brazil, its incidence is higher, and because of the importance of exploring another possible etiological or risk factor, that is, EBV. HPV in penile cancer has been recognized in almost 50% of the cases. In contrast, the presence and biological and clinical relevance of EBV in penile cancer have not been sufficiently studied. However, it is necessary to review the scope of the findings and analyze the concomitant presence of both viruses and the clinical and pathological characteristics of the patients, in order to define the possible relationship of EBV in penile cancer. In this sense, it is necessary to consider the following observations.

**Title**

“HIGH PREVALENCE OF HPV WITH OVEREXPRESSION OF p16 INK4A PROTEIN AND CO-INFECTION WITH EBV IN PENILE CANCER – A SERIES OF CASES FROM BRAZIL AMAZON”. The title suggests that the authors found a higher prevalence of HPV than the one reported worldwide and that there is a concomitant presence of EBV infection in HPV positive cases. However, the data obtained does not reflect this. I suggest that the title should be modified to reflect in the best way the scope of the study and the results obtained.

*We agree and have rewritten the title.*

***PRESENCE OF HPV WITH OVEREXPRESSION OF p16*** *^INK4A^* ***PROTEIN AND EBV INFECTION IN PENILE CANCER – A SERIES OF CASES FROM BRAZIL AMAZON***

**Abstract**

Line 27. It reads “Epstein Baar”; it should read “Epstein-Barr”.

*We have corrected in the texts. (Line 29)*

Line 57. It reads “Con-infection”; it should read “co-infection”.

*We have corrected in the texts. (Line 51-52)*

The conclusion states that “p16 INK4a positivity presented a high correlation to HPV 16 DNA detection, reinforcing its use as a surrogate marker for HPV-driven cancers.” This is not a novel finding. Thus, it is necessary to rewrite the conclusion in the abstract and the text after analyzing the group of concomitant HPV and EBV infection and EBV alone.

*We totally agree and have brought the necessary changes. (Line 51-52)*

**Introduction**

Line 56-58. It is necessary to update the references about epidemiological data of penile carcinoma, since the only reference is from 2010, and there are recent works on this issue.

*We have brought the necessary changes. (Line 55-57)*

Line 69-70. Concerning HPV prevalence in penile carcinoma, it is necessary to complement and or compare the most recent data worldwide (Bruni 2017; Alemany 2016; Olesen 2019).

*We have brought the necessary changes. (Line 69-71)*

Line 80-86. It is important to describe the oncogenic role of EBV in cancer, as currently known, and the cell types affected by such viruses. The relationship between EBV and HPV in carcinogenesis is described in various papers; one of them is a review by Guidry and Scott (doi:10.1016/j.virusres.2016.11.002.). It is necessary to expand the references about the oncogenic role of EBV in epithelial carcinogenesis.

*We have corrected accordingly. (Line 81-88)*

Line 87-88. It is necessary to describe in a better way the rationale or the relevance of the work, emphasizing the study of EBV and HPV, and the few studies specifically in penile carcinoma.

*We totally agree and have brought the necessary changes. (Line 89-90)*

**Materials and Methods**

Line 112-113. Specify the size of the b-globin product.

*We have provided in the text. (Line 115-117)*

Line 126. What is the reason to do specific E7 HPV 16 and 18? The results and discussion about this detection are not mentioned. Besides, the genotyping by PapilloCheck test was used. The rationale to detect E7 is not indicated.

*Reply*

*As PapilloCheck is a very expensive test, we have chosen to primarily screen the presence of HPV followed by typing specifically for HPV16 and HPV18. All of the samples that were negative for HPV16 and HPV18 were screen by PapilloCheck test to reduce the cost of typing.*

Line 171. It is necessary to indicate why only 21/47 samples were analyzed.

*Reply*

*We believe that there is misunderstanding here. We did already explain in the text. 21 samples were not available for pre-analytical analysis due to poor quality keeping of the samples.*

**Results**

Tables 1 and 2 could be merged.

*We do agree but merging both tables 1 and 2 will be heavy and confusing.*

Line 210-211. In patients with EBV and HPV co-infection, the relationship with p16 overexpression should be described.

*We have provided in the text. (Line 223-224)*

Table 3. Include the information about the expression of p16.

*Reply*

*As we did for only 26 samples, we did not include in the Table 3 to avoid confusion, but we describe in text.*

Line 215-216. It would be important to evaluate the presence of HPV newly (with a different method, if possible) in the positive cases of p16, which were initially identified as negative for HPV DNA.

*Reply*

*The four samples that were positive for p16 and negative for HPV were again tested for the presence of HPV16 and HPV18 by real-time PCR and continued to be negative.*

Line 219-220. Smoking and HPV presence are relatively excluding factors, at least in HNCSS. The relationship between overexpression of p16 and smoking was identified; however, it was not so with the presence of HPV. The analysis was made with all genotypes identified (low and high risk). What is the relationship between smoking and high-risk HPV presence?

*Reply*

*We have rewritten in the result section to avoid confusion. There was no relationship between smoking and high-risk HPV presence. As it can be seen that the driving force for the presence of p16 is mostly dependent on the presence of HPV and independent of the smoking status. (Line 232-238)*

Line 223-228. It is necessary to describe the survival analysis of the group with co-infection of HPV and EBV since the analysis was only made individually, and an important issue (since the title of the study) is the co-infection of EBV and HPV. The result should be discussed.

*Thank you for pointing this lack. We have performed the analysis and is shown in Figure 2D. The comparison showed no difference P _log rank_=0.318. We also included a S1 Table showing the infection status of the deceased and survived patients. (Line 240-241)*

Table 4. This table could be simplified by removing the percentages (leaving the frequencies) and adding the EBV presence in the header row and respective analysis.

*We have brought the necessary changes.*

**Discussion**

Line 251. The metastasis analysis should be extended. This issue has been addressed in head and neck carcinomas HPV negative versus positive, and also in nasopharyngeal carcinomas EBV positive, as well as prognosis factors associated.

*Reply*

*As our study is only focused on penile cancer, we did not compare with other types of cancers as we believe that the developing mechanisms could be different. For this reason, we did not extend the discussion.*

Line 252-253. The result about HPV in young men contrasts with a recent article (doi.org/10.1186/s12879-019-4696-6), reporting that at least 50% of men under 45 years have HPV. It is important to extend the discussion about the characteristics of penile cancer in young men and contrast the results with other reports.

*Reply*

*We agree to disagree because of the sample size which makes it difficult to compare. In the study stated, there were only 8 patients under 45 years old and four of them were positive for HPV. In our study we had 13 patients under 45 years old and five (38%) were positive for HPV.*

Line 258. Change the capital letters in the author's name cited.

*We have corrected in the texts. (Line 259)*

Line 268. According to the data in Table 4, tumor grade III is associated with HPV presence. Improve the description of this finding and mention the concordance with previous works.

*We have added one more reference. (Line 292)*

Line 275-276. How could you explain the relationship between phimosis and overexpression of p16?

*We have provided in the discussion. (Line 300-312)*

Line 279-285. The relationship between EBV and HPV should be expanded. Although the relation between EBV and HPV in penile cancer is incipient, the relationship is described in another HPV-related cancer. Several articles have described the association between expression of p16 and EBV in other neoplasms (Doi: 10.1002/hed.24258. doi.org/10.1016/j.ijrobp.2017.06.1473. doi.org/10.3389/fonc.2018.00113), and this is not described in the discussion.

*Reply*

*As cited previously (Answer Line 252). We have focus mainly on penile cancer. Considering that in other studies with penile cancer the HPV and EBV status were not predictive of outcome and we believe that the developing mechanisms could be different for another cancer.*

Line 289-290. The absence of HPV 18 in penile carcinoma is consistent with other works. (doi.org/10.1186/s12879-019-4696-6), and in contrast with the findings in other regions (DOI: 10.1002/jmv.20703).

*We have included in the text. (Line 326-329)*

Line 295. Describe more broadly the possible mechanisms for the synergistic effect of HPV 6 and EBV.

*We included in the text. (Line 333)*

Line 302-303. What other genotypes (different to HPV 16) were present in the p16 positives cases?

*We have described in the result section. (Line 229-229)*

Line 304. Change the capital letters of the author's name cited.

*We have corrected in the texts. (Line 342)*

Line 313, Figure 2. The correlation between p16 overexpression and survival has been addressed in other neoplasms. The authors should review the impact of the clinical stage on survival. Did you consider the local and advanced stages for survival analysis?

*Reply*

*We have included in the text. However, we did stratify the patients according to the local and advanced stages as our sample size is small. And stratification will further reduce the sample and will not have any power to detect any difference according to our statistician. (Line 350-351)*

Finally, the authors should discuss the limitations of the study. For example, the number of p16 samples analyzed, if the size (3-5 mm) of samples analyzed was adequate for the detection of the viruses, p16, and pathological characteristics.

*We have described in the discussion section. And we also described in the materials and methods section and Supporting Information (S2 PROTOCOL - Immunohistochemistry for p16^INK4a^).*

One issue not mentioned is the presence of HPV without relation to penile carcinogenesis. Therefore, it is important to describe the p16 overexpression as a surrogate biomarker of carcinogenesis induced by high-risk HPV. The analysis of EBV dependent and independent of HPV should be extended and, consequently, the discussion.

*We have described in the discussion section.*

Sincerely yours,

Yours faithfully,

Valquiria do Carmo Alves Martins

*On behalf of all authors*
